# Diagnostic accuracy of artificial intelligence based on imaging data for predicting distant metastasis of colorectal cancer: a systematic review and meta-analysis

**DOI:** 10.3389/fonc.2025.1558915

**Published:** 2025-05-12

**Authors:** Lulin Chen, Fei Xu, Lujiao Chen

**Affiliations:** ^1^ Postgraduate Affairs Department, Zhejiang Chinese Medical University, Hangzhou, Zhejiang, China; ^2^ Department of Ultrasound, Affiliated Hospital of Shaoxing University, Shaoxing, Zhejiang, China; ^3^ Department of Radiology, Shaoxing People’s Hospital, Shaoxing, Zhejiang, China

**Keywords:** colorectal cancer, distant metastasis, CT, MR, ultrasound, artificial intelligence, deep learning, machine learning

## Abstract

**Background:**

Colorectal cancer is the third most common malignant tumor with the third highest incidence rate. Distant metastasis is the main cause of death in colorectal cancer patients. Early detection and prognostic prediction of colorectal cancer has improved with the widespread use of artificial intelligence technologies.

**Purpose:**

The aim of this study was to comprehensively evaluate the accuracy and validity of AI-based imaging data for predicting distant metastasis in colorectal cancer patients.

**Methods:**

A systematic literature search was conducted to find relevant studies published up to January, 2024, in different databases. The quality of articles was assessed using the Quality Assessment of Diagnostic Accuracy Studies 2 tool. The predictive value of AI-based imaging data for distant metastasis in colorectal cancer patients was assessed using pooled sensitivity, specificity. To explore the reasons for heterogeneity, subgroup analyses were performed using different covariates.

**Results:**

Seventeen studies were included in the systematic evaluation. The pooled sensitivity, specificity, and AUC of AI-based imaging data for predicting distant metastasis in colorectal cancer patients were 0.86, 0.82, and 0.91. Based on QUADAS-2, risk of bias was detected in patient selection, diagnostic tests to be evaluated, and gold standard. Based on the results of subgroup analyses, found that the duration of follow-up, site of metastasis, etc. had a significant impact on the heterogeneity.

**Conclusion:**

Imaging data images based on artificial intelligence algorithms have good diagnostic accuracy for predicting distant metastasis in colorectal cancer patients and have potential for clinical application.

**Systematic review registration:**

https://www.crd.york.ac.uk/PROSPERO/, identifier PROSPERO (CRD42024516063).

## Introduction

Colorectal cancer (CRC) ranks third in terms of frequency and has the third-highest occurrence rate and second-highest death rate globally (1). The primary reason for mortality in patients with colorectal cancer is distant metastasis. Even with surgical removal, approximately 50% of patients experience metastasis, and around 25% of colorectal cancer patients already have distant metastasis when initially diagnosed (2, 3). The primary sites of metastasis include the liver, lungs, peritoneum, and peripheral lymph nodes. Additionally, there may be localized metastases to the bone, adrenal glands, ovaries, brain, pancreas, and spleen (4). The five-year survival rate for patients diagnosed with stage I-II colorectal cancer is between 88% and 95%. In contrast, patients with metastatic colorectal cancer have a survival range of 3 months to 5 years, with around 60% of them dying within 1–2 years (5). Hence, doing an early evaluation and forecast of distant metastases in patients with colorectal cancer is advantageous for enhancing prognostic outcomes and mitigating the possible hazards linked to aggressive multimodal therapy (6).

Medical imaging is frequently employed to visualize the dissemination of tumors and measure their severity, offering significant data for diagnosis, staging, and treatment planning. For instance, contrast-enhanced ultrasound (CEUS), multidetector computed tomography (MDCT), magnetic resonance imaging (MRI), and fluorodeoxyglucose (FDG) positron emission tomography (PET)/CT exhibit a sensitivity and specificity of 80% and 97% respectively in the detection of liver metastases from colorectal cancer (7). Nevertheless, the task of accurately and promptly diagnosing medical conditions using imaging techniques is arduous because of the imbalance between the number of doctors and patients and the complexity of radiologic diagnosis.

Artificial Intelligence (AI) has become an essential component of healthcare in recent years, utilizing algorithms, machine learning, computers, and data science. Furthermore, using AI has led to a rise in AI-driven studies, as AI can measure elements of imaging that are imperceptible to the human eye. This enables the early detection of tumors or the spread of cancer cells in imaging images (8). Artificial intelligence (AI), which encompasses deep learning (DL), refers to the programming of computers to imitate human intelligence. Semi-automated AI involves using conventional machine learning methods, including radiomics, in which the radiologist is required to carry out specific preprocessing tasks on the picture to ensure its compatibility with the algorithm. Neural networks are a specific type of deep learning model that imitates the functioning of the human visual cortex. The neural network layer comprises neurons that identify various image characteristics through edge, color, and texture filters (9–11). Artificial intelligence-driven radiomics applies sophisticated computational methods to extract several investigator-defined characteristics from medical pictures (12). Although radiomics models have been somewhat successful in predicting CRC lymph node metastasis, previous studies conducted by Ding et al. and Wang et al. have demonstrated that deep learning algorithms can detect more nuanced patterns that are not discernible by conventional radiological and statistical techniques (13, 14).

There is currently a lack of effective methods for predicting the distant spread of colorectal cancer, which could help create personalized treatment plans for high-risk patients undergoing extensive surgery. AI technology has the potential to detect which colorectal cancer patients are in danger of developing distant metastasis before it occurs. Despite numerous research studies on the use of AI in evaluating colorectal cancer metastasis, a dearth of recent systematic reviews thoroughly examine the effectiveness of AI-based medical imaging in accurately predicting outcomes. This study aims to conduct a systematic review and meta-analysis to analyze and summarize the existing research data using AI-assisted medical imaging, specifically CT, MRI, and ultrasound, to assess colorectal cancer metastasis. The study also aims to evaluate these imaging techniques’ diagnostic accuracy, sensitivity, and specificity. This will enable clinicians to forecast patients’ prognostic information better and choose treatment plans more precisely.

## Methods

This systematic review followed the Preferred Reporting Items for Systematic Reviews and Meta-Analyses of Diagnostic Test Accuracy Studies (PRISMA-DTA) guidelines (15). This study is registered with the Prospective International Registry of Systematic Evaluation (PROSPERO) (ID: CRD42024516063).

### Search strategies and literature screening

We conducted a comprehensive search of various databases, including PubMed (Medline), Embase, the Cochrane Library, and Web of Science, to identify studies related to the topic up to January 31, 2024. We used a combination of Medical Subject Headings (MeSH)/Emtree Glossary and free-form words as search terms for titles and abstracts. Additionally, we manually searched the reference lists of relevant studies, reviews, and meta-analyses to ensure that no potential research literature was missed. We did not restrict our search to any particular year of publication but only included studies published in English. The search keywords we used were “colorectal cancer,” “metastasis,” “artificial intelligence,” “deep learning,” “machine learning,” and “radiomics.” For more information on the search keywords used for each database, see **Supplementary Material 1**.

All studies retrieved from relevant databases were collated in Endnote X9.3.3 (Clarivate Analytics, London, UK), and duplicates were removed. Two independent researchers independently screened the titles and abstracts of all retrieved studies, eliminated articles that did not meet eligibility criteria, and assessed the full text for final inclusion. Any disagreements in the screening were resolved through discussion or consultation with a third researcher.

All studies retrieved from relevant databases were collated in Endnote X9.3.3 (Clarivate Analytics, London, UK), and duplicates were removed. Two independent researchers independently screened the titles and abstracts of all retrieved studies, eliminated articles that did not meet eligibility criteria, and assessed the full text for final inclusion. Any disagreements in the screening were resolved through discussion or consultation with a third researcher.

### Inclusion and exclusion criteria

Articles meeting the following criteria were included: (1) inclusion of patients with histopathologic diagnosis of colorectal cancer; (2) development or use of artificial intelligence algorithms based on imaging data such as CT, MRI, or ultrasound to assess distant metastasis; (3)research employing radiomics, machine learning, or deep learning methodologies for the prediction of metastasis;(4) studies detailing sensitivity, specificity, or receiver operating characteristic (ROC) curve analyses evaluating the effectiveness of AI-based imaging models in predicting the reliability of distant metastasis in colorectal cancer; (5) the study was an observational study (retrospective or prospective), randomized or non-randomized controlled trial; (6) language restriction to English.

Studies were excluded based on the following criteria: (1) case reports, reviews, review articles, editorials, letters, and conference abstracts; (2) animal studies; (3) studies that were not relevant to this study; (4) studies not based on imaging data; and (5) research relying solely on conventional imaging interpretation, excluding artificial intelligence components. By applying the above inclusion and exclusion criteria, we aimed to ensure the studies’ quality and reliability and minimize potential biases and errors.

### Data extraction and quality assessment

Two researchers performed data extraction independently, and a third researcher resolved their differences. The results of the data extraction included the following: (1) last name of the first author; (2) year of publication; (3) source of participants, country; (4) type of study; (4) number of patients, age; (5) Sample grouping method and model validation method; (6) duration of follow-up; (7) site of metastatic tumor; (8) sample size of metastasis; (9) type of imaging data; (10) input data; (11) selection of model features; (12) specific algorithm of artificial intelligence used for constructing the model; and (13) area under the receiver operating curve (AUC) of the subjects and other parameters. The following data were extracted from the included studies: the data collected were four-cell tabulated data (2 × 2), including true positive (TP), true negative (TN), false positive (FP), and false negative (FN). When comparing the diagnostic performance of different algorithms for the same sample, the algorithm that produced the best classification results was selected. If there were no sensitivities or specificities in a study, we used Engauge Digitizer (version 12.1, Mark Mitchell) to calculate sensitivities and specificities at the maximum of the Youden Index based on the receiver operating characteristic (ROC) curves from the article. If there were more than two models for the same group of patients in a study, the model with the higher AUC value was included in our meta-analysis.

The methodological quality and risk of bias of the included studies were assessed by the Quality Assessment of Diagnostic Accuracy Studies (QUADAS-2) (16), which assessed a total of four domains, including the selection of cases, the experiments to be evaluated, the gold standard, and the case flow and progression. All components are assessed in terms of risk of bias, and landmark questions are included in the risk of bias judgment, and according to the answer of “yes,” “no,” or “not sure” to the relevant landmark question included in each component, the bias can correspond to the risk of bias. The risk of bias was assessed as “low,” “high,” or “uncertain” according to the “yes,” “no,” or “uncertain” answers to the relevant landmark questions included in each section. Any disagreement was resolved by consensus. The evaluation was performed using Revman 5.3 (Cochrane Collaboration, UK).

### Statistical analysis

Stata 14.2 (StataCorp LP, College Station, TX, USA) was used for the data analysis. Due to the significant heterogeneity of this study, we combined the relevant diagnostic accuracy indicators, including sensitivity, specificity, diagnostic odds ratio (DOR), NLR, and PLR, using a bivariate random-effects model. The model’sAUC was calculated using the summary receiver operating characteristic(SROC). A threshold effect test was conducted using Meta-disc version 1.4 (Hospital Ramon y Cajal and Complutense University of Madrid, ESP). The presence or absence of a threshold effect was determined by calculating the Spearman’s correlation coefficient between the logarithm of sensitivity and the logarithm of (1-specificity). A strong positive correlation indicated the presence of a threshold effect. The heterogeneity of the results of the included studies was assessed using Cochran’s Q test, combined with I^2^ statistics. If heterogeneity was evident, factors controlling model accuracy were identified by meta-regression using pre-specified covariates: imaging modality, study setting, validation method, site of transfer, type of AI algorithm, and so on. Deek funnel plots assessed the publication bias of the included studies, and sensitivity analyses were used to assess the stability of the results. Post-test probabilities were calculated to assess clinical utility, and Fagan plots were drawn. The combined effect value of multiple studies was statistically significant if P ≤ 0.05.

## Results

### Literature search

**Figure 1:**
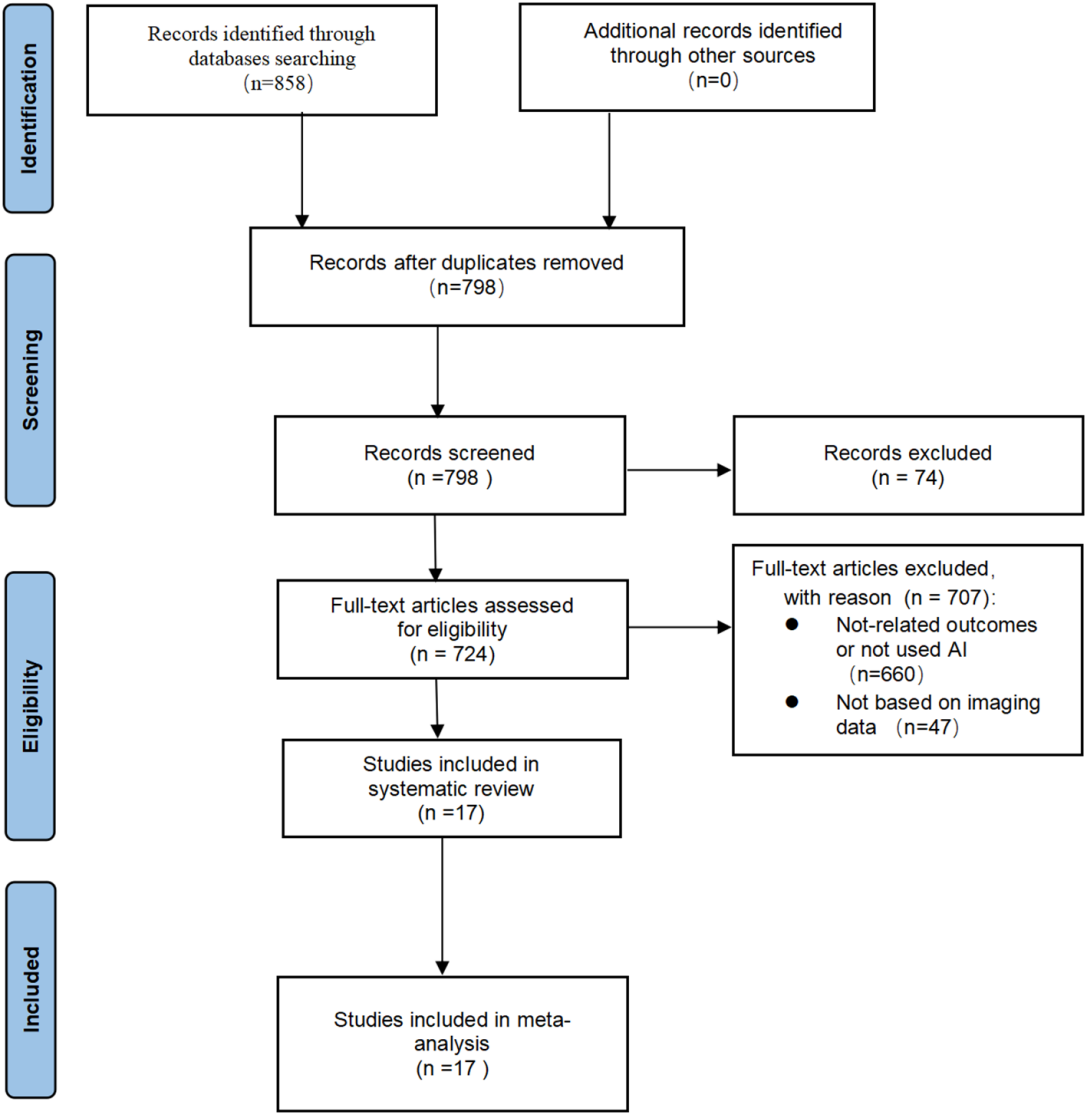
PRISMA flowchart of literature screening.

Initially, 858 articles (141 in PubMed, 115 in Embase, 32 in Cocharne Library, 570 in Web of Science) were identified through PubMed, Embase, Cocharne Library, and Web of Science databases using keywords. A total of 60 duplicates were removed, and 74 records were excluded after screening titles and abstracts because they were abstracts, conference proceedings, letters, reviews, meta-analyses, or case reports. The remaining 724 studies were reviewed in full text and screened against the inclusion and exclusion criteria, resulting in the inclusion of 17 studies. A summary of the PRISMA flowchart is shown in (**Figure 1**).

### Literature quality assessment

**Figure 2:**
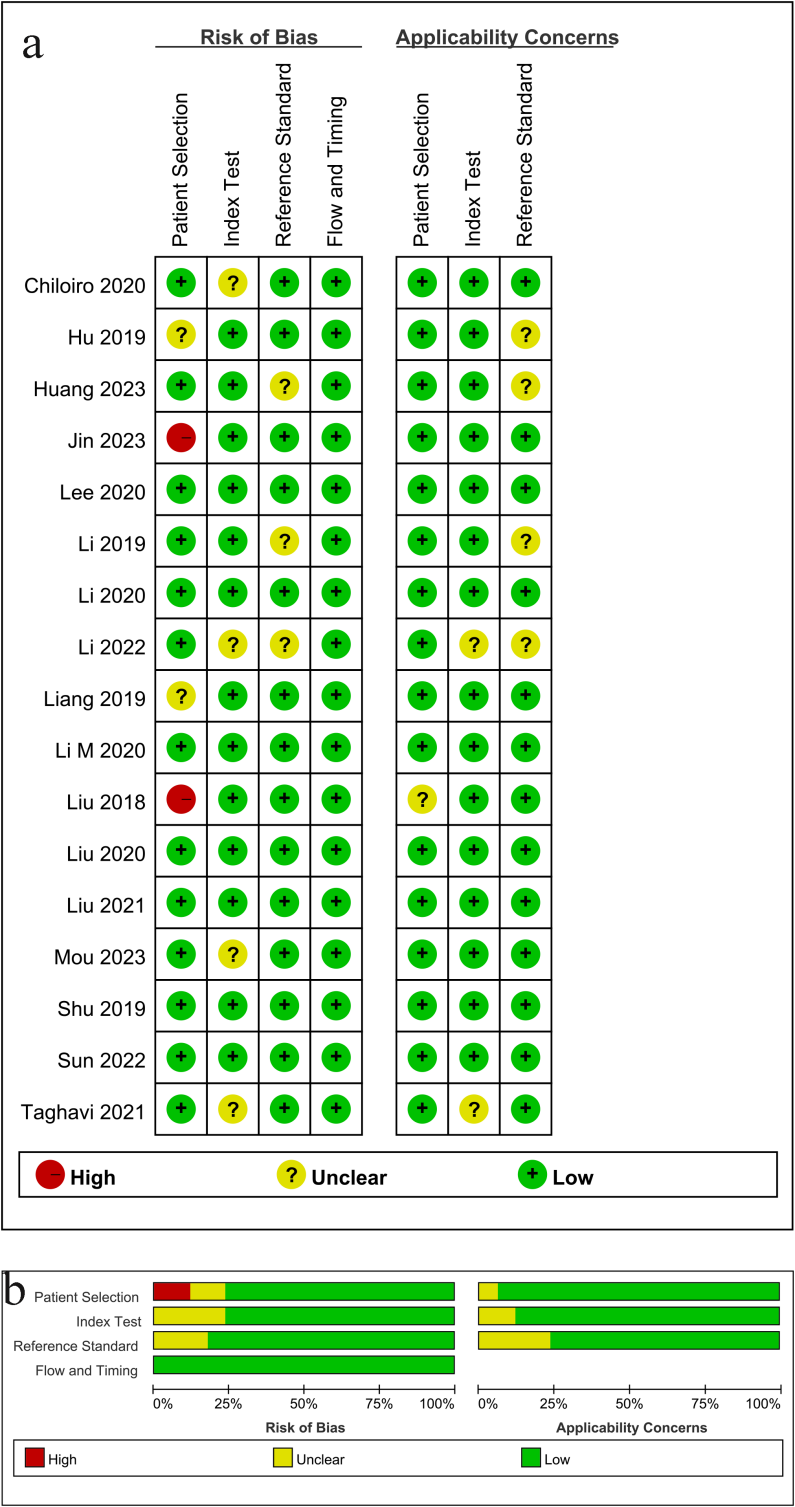
Risk of bias and applicability concerns according to Quality Assessment of Diagnostic Accuracy Studies-2 tool: **(a)** per study assessment **(b)** per domain summary.

QUADAS-2 was used to examine the risk of bias and applicability issues of the included studies (**Figure 2**). Regarding patient selection, two studies showed a high risk of bias because they did not avoid inappropriate patient exclusion. Four studies had an unclear risk of bias in “trials to be evaluated” because they did not clearly describe how their index test was performed and interpreted and did not use pre-specified thresholds. Three studies had an unclear risk of bias in the “gold standard” domain because the blinding setting was not considered. Finally, regarding the risk of bias in the area of “process and progress,” almost all studies were considered to have a low risk of bias. Overall, concerns about applicability were low.

### Characteristics of included studies

These 17 studies (17–33) included 5474 patients, with an age range of 19–89 years for inclusion and a follow-up time range of 24–60 months, with eight studies not mentioning the follow-up time. Of the included studies, 14 were conducted in China, one in Italy, one in New Zealand, and one in South Korea. All studies were retrospective except for one prospective study (24). Of these studies, 14 were single-center, and the remaining three were multicenter (22, 27, 32). Almost all of the models in these studies were internally validated, with only three studies being externally validated (23, 27, 32). The algorithms in these studies essentially randomized patients into training and validation groups in a 7:3 ratio. **Table 1** further summarizes the characteristics of the included studies and the patient statistics.

**Table 1:** Baseline data from included studies.

Studies	Design	Data source	Country	Number of Patients	Age (years)	Sample grouping method	Validation method	Follow-up (months)
Li, 2019 (17)	Retrospective	Single center	China	48	63.33 ± 11.21	1(TC):1(VC)	Cross-validation	NR
Liang, 2019 (18)	Retrospective	Single center	China	108	54.5± 10.9	54(LM):54(Nonmetastasis)	Cross-validation	24
Shu, 2019 (19)	Retrospective	Single center	China	192	NR	7(TC):3(VC)	Cross-validation	NR
Lee, 2020 (20)	Retrospective	Single center	Korea	2019	63.1 ± 9.5	7(TC):3(VC)	Cross-validation	60
Li, 2020 (21)	Retrospective	Single center	China	100	59.5 (52.0–68.5)	8(cross-validation set):2(test set)	Cross-validation	NR
Taghavi, 2021 (22)	Retrospective	Multicenter	The Netherlands	91	64 ± 11	split into a training set (n=70) and independent validation set (n=21)	Cross-validation	24
Li, 2022 (23)	Retrospective	Single center	China	323	61 (53-69)	171(TC);77(IVC);75(EVC)	external validation	NR
Sun, 2022 (24)	prospective	Single center	China	150	Range: 51–62	50(initial LM):50(follow-up LM):50(Nonmetastasis)	train-test split	24
Jin, 2023 (25)	Retrospective	Single center	China	614	58.0 (49.3, 68.0)	7(TC):3(VC)	Cross-validation	NR
Li M, 2020 (26)	Retrospective	Single center	China	148	59.7 ± 11.7	7(TC):3(VC)	train-test split	36
Liu, 2021 (27)	Retrospective	Multicenter	China	235	54.82± 10.85	170(primary cohort);65(EVC)	external validation	36
Liu, 2019 (28)	Retrospective	Single center	China	177	64 (21–88)	7(TC):3(VC)	train-test split	NR
Chiloiro, 2020 (29)	Retrospective	Single center	Italy	213	64 (26-83)	90%(Training and cross-validation data):10%(Testing data)	Cross-validation	60
Hu, 2019 (30)	Retrospective	Single center	China	194	58.6 ± 10.73 y	7(TC):3(VC)	Cross-validation	24
Liu, 2020 (31)	Retrospective	Single center	China	169	57.0 ± 10.6	7(TC):3(VC)	train-test split	NR
Huang, 2023 (32)	Retrospective	Multicenter	China	454	55.47 ± 11.43	8(TC):2(IVC);EVC(81)	external validation	36
Mou, 2023 (33)	Retrospective	Single center	China	239	Range: 19–89	7(TC):3(VC)	Cross-validation	NR

EVC, independent external validation cohort; LM, liver metastasis; NR, not reported; IVC, internal validation cohort; TC, training cohort; VC, validation cohort.

Distant metastasis of colorectal cancer was reported in all studies, and liver metastasis was the most common site of metastasis with a metastasis rate of 10.85% (594/5474). In addition, lung metastasis was 2.43% (133/5474), peritoneal metastasis was 0.05% (3/5474), and bone metastasis was 1.02% (56/5474). **Table 2** summarizes these findings.

**Table 2:** Summary analysis of distant metastases by site.

Studies	Total number of patients	distant metastatic site	Number of metastasis	Number of hepatic metastasis	Number of pulmonary metastasis	Peritoneal metastasis	Bone metastasis	Synchronous metastasis
Li, 2019 (17)	48	Hepatic	24	24	NR	NR	NR	NR
Liang, 2019 (18)	108	Hepatic	54	54	NR	NR	NR	NR
Shu, 2019 (19)	192	Hepatic	111	111	NR	NR	NR	NR
Lee, 2020 (20)	2019	Hepatic	100	100	NR	NR	NR	NR
Li, 2020 (21)	100	Hepatic	50	50	NR	NR	NR	NR
Taghavi, 2021 (22)	91	Hepatic	24	24	NR	NR	NR	NR
Li, 2022 (23)	323	Hepatic	23	23	NR	NR	NR	NR
Sun, 2022 (24)	150	Hepatic	100	100	NR	NR	NR	NR
Jin, 2023 (25)	614	Bone	53	NR	NR	NR	53	NR
Li M, 2020 (26)	148	Multiple	51	15	24	NR	NR	12
Liu, 2021 (27)	235	Multiple	68	NR	NR	NR	NR	NR
Liu, 2019 (28)	177	Multiple	59	27	16	3	3	11
Chiloiro, 2020 (29)	213	Multiple	72	NR	NR	NR	NR	NR
Hu, 2019 (30)	194	pulmonary	93	NR	93	NR	NR	NR
Liu, 2020 (31)	169	Hepatic	32	32	NR	NR	NR	NR
Huang, 2023 (32)	454	Multiple	121	NR	NR	NR	NR	NR
Mou, 2023 (33)	239	Hepatic	34	34	NR	NR	NR	NR

NR, not reported.

CT and MRI were the imaging modalities used in most of the studies, with eight studies each using them and the remaining one using US (33). Due to the high dimensionality and complexity of imaging data using different sequences, feature selection reduces the computational power required to perform such complex analyses.LASSO is often used for feature selection (18, 21, 24, 26, 30–33). Other methods often used for dimensionality reduction include analysis of variance (ANOVA) and Mann-Whitney U test (MW) (19), principal component analysis (PCA) (20), recursive feature elimination (RFE) (23), and Pierce’s correlation coefficient (25, 28, 29). For the problem of a very unbalanced dataset, Lee S and Li Y et al. (20, 23) researchers used the Synthetic Minority Oversampling Technique (SMOTE) to increase the number of minority samples in the dataset. Jin J et al. (25) balanced the positive and negative samples by reducing the samples and using Min Max to normalize the feature matrix. Different authors used different artificial intelligence algorithms for modeling, and all algorithms showed good predictive results in validation (AUC > 0.7). Among the 17 eligible studies, the commonly used algorithms are Random Forest (RF), Logistic Regression (LR), Support Vector Machines (SVM), Convolutional Neural Networks (CNN), Multi-Layer Perceptron Networks (MLP), etc., and the detailed information is shown in **Table 3** and **Supplementary Material 2**. 3 of the included studies (20, 25, 27) developed neural networks. Lee S et al. (20) developed the neural networks by utilizing a pre-trained convolutional neural network VGG16 for feature extraction of images, which did not require further training. For model construction, Jin J et al. (25) proposed an artificial neural network model (ANNM). The ANN algorithm can detect complex nonlinear relationships between dependent and independent variables and does not require much formal statistical training. It can provide a variety of training algorithms to improve the model’s performance. It is worth mentioning that the CNN proposed by Liu X et al. (27) is based on the residual structure, which can solve the problem of gradient vanishing, and at the same time, in order to make the dataset contain complete tumor information, the use of early stopping and appropriate dropout can effectively improve the robustness of the model. The results showed that the AUC of the CNN model in the validation cohort was 0.892.

**Table 3:** Basic features of predictive models for imaging data based on artificial intelligence algorithms.

Studies	Image	Input data	Feature selection	AI algorithm	AUC	Accuracy	Specificity	Sensitivity	NPV	PPV
Li, 2019 (17)	CT	Portal venous phase images	heterogeneity;entropy,;energy of vertical wavelet image; diameter of tumor	RELIEFF, SVM	0.96	0.91	0.95	0.86	NR	NR
Liang, 2019 (18)	MR	high-resolution oblique axial (perpendicular to the long axis of the tumor) T2WI without fat saturation; axial three-dimensional liver acquisition with volume acceleration multienhanced MR images	Firstorder;GLCM;GLRLM;GLSZM;	SVM, LR	0.87	0.80	0.76	0.83	NR	NR
Shu, 2019 (19)	MR	T2WI	GLCMEntropy_ALLDirection_offset1_SD;GLCMEntropy_ALLDirection_offset1; ShortRunEmphasis_ALLDirection_offset7_SD;ShortRunEmphasis_angle35_offset7; LongRunEmphasis_angle45_offset7;GreyLeveLNonuniformity_ALLDirection_offset7_SD;RunLengthNonuniformity_ALLDirection_offset4_SD	LASSO, PCA, LR	0.89	0.92	0.79	0.95	NR	NR
Lee, 2020 (20)	CT	non-contrast abdominal CT scan image	Clinical features(age,gender, T stage and N stage);imaging features(sequential summation of PC1 to PC10)	VGG, PCA, LR, RFC	0.75	NR	NR	NR	NR	NR
Li, 2020 (21)	CT	Portal venous phase images	Firstorder;GLDM;GLCM;GLRLM;GLSZM;IDMN	LASSO, SVM, RFC, GBDT, LR, MLP, SCLF	0.90 ± 0.02	NR	0.79 ± 0.04	0.85 ± 0.02	0.85 ± 0.02	0.81 ± 0.03
Taghavi, 2021 (22)	CT	Portal venous phase images	Firstorder;GLCM;GLRLM;GLSZM; MGTDM;GLDM	RF, DT, ML	0.95	NR	NR	NR	NR	NR
Li, 2022 (23)	CT	Portal venous phase images	Three original image features, two wavelet image features and one LoG image feature.	SMOTE, MLP, RFE, SVM	0.85	0.74	0.77	0.81	0.79	0.53
Sun, 2022 (24)	CT	Portal venous phase images	NR	LASSO, LR, LD, KNN, NB, DT, SVM	NR	0.78	0.76	0.81	NR	NR
Jin, 2023 (25)	MR	DWI	GLCM	RF, DT, ANN, SVM	0.93	NR	NR	NR	NR	NR
Li M, 2020 (26)	CT	Portal venous phase images	MaxIntensity; RelativeDeviation;Inertia_AllDirection_offset7_SD	LASSO, LR	0.84	0.98	0.70	0.92	NR	NR
Liu, 2021 (27)	MR	T2WI; DWI	prognostic-related imaging information	ResNet18	0.93	NR	0.89	0.86	NR	NR
Liu, 2019 (28)	MR	oblique axial T2WI images	mrN staging, SphericalDisproportion, CA199, GLCMEntropy_ angle90_offset7, GLCMEnergy_angle135 _offset7, Inertia_AllDirection_offset1_SD, CEA,SurfaceArea,GLCMEntropy_ angle0_offset4, and gender.	RF, LR	0.83	0.87	0.94	0.72	0.87	0.87
Chiloiro, 2020 (29)	MR	T2-weighted fast spin-echo 3D highresolution images	medianFD 30,60.delta; F_szm.lzlge 1.1.delta; F_morph.pca.flatness.pre; F_cm.clust.prom 0.6.pre	LR	NR	0.81	0.86	0.71	0.86	0.71
Hu, 2019 (30)	CT	Reconstructed images	Hist;GLCM;GLRLM;FD;LoG	LASSO, LR	0.93	0.88	0.91	0.85	0.84	0.93
Liu, 2020 (31)	MR	high-resolution T2WI	exponential_ngtdm_Coarseness; exponential_glszm_La … ighGrayLevelEmphasis; wavelet-LLH_firstorder_Median’ wavelet-LHH_gldm_DependenceVariance; wavelet-LLH_glcm_ClusterShade	LASSO	0.92	0.82	0.79	0.90	0.96	0.60
Huang, 2023 (32)	MR	T2WI, DWI, CE-T1WI	GLCM;GLRLM;GLSZM; GLDM; NGTDM	LASSO, LR, ComBat	0.89	0.81	0.81	0.82	0.93	0.59
Mou, 2023 (33)	Ultrasound	ultrasound images	Hist;GLCM; GLSZM; Firstorder;	LASSO, LR	0.92	0.77	0.73	0.96	NR	NR

AI, artificial intelligence; ANN, artificial neural network; AUC, area under the curve; CE-T1WI, contrast-enhanced T1-weighted imaging; CNN, convolutional neural network; CT, computed tomography; DT, decision tree; DWI, diffusion-weighted imaging; FD, fractal dimension; GBDT, gradient boosting decision tree; GLCM, gray-level co-occurrence matrices; GLDM, gray level dependence matrix; GLRLM, gray-level run length matrix; GLSZM, gray-level size zone matrix; Hist, histogram; KNN, k-nearest neighbor; Lasso, least absolute shrinkage and selection operator; IDMN, inverse difference moment normalized; LR, logistic regression; LD, linear discriminant; LOG, laplacian of gaussian filter; MRI, magnetic resonance imaging; ML, machine learning; MLP, multilayer perceptron; NB, naive Bayesian; NGTDM, neighboring gray tone difference matrix; NPV, negative predictive value; NR, not reported; PC, principal component; PCA, principal component analysis; PPV, positive predictive value; RFE, recursive feature elimination; RF, random forest; RFC, random forest classifier; RLM, run length matrix; SVM, support vector machine; SCLF, stacking classifier; SMOTE, synthetic minority oversampling technique; T2WI, T2-weighted imaging; VGG, **v**isual **v**eometry **v**roup.

In machine learning models, a hyperparameter is an adjustable parameter that needs to be initialized before training the model and is critical to its performance. Taghavi M et al. (22) used Bayesian Hyperparameter Optimization. This iterative search procedure uses simpler machine learning algorithms to find the highest-performing hyperparameter combinations.

### Meta-analysis

**Figure 3:**
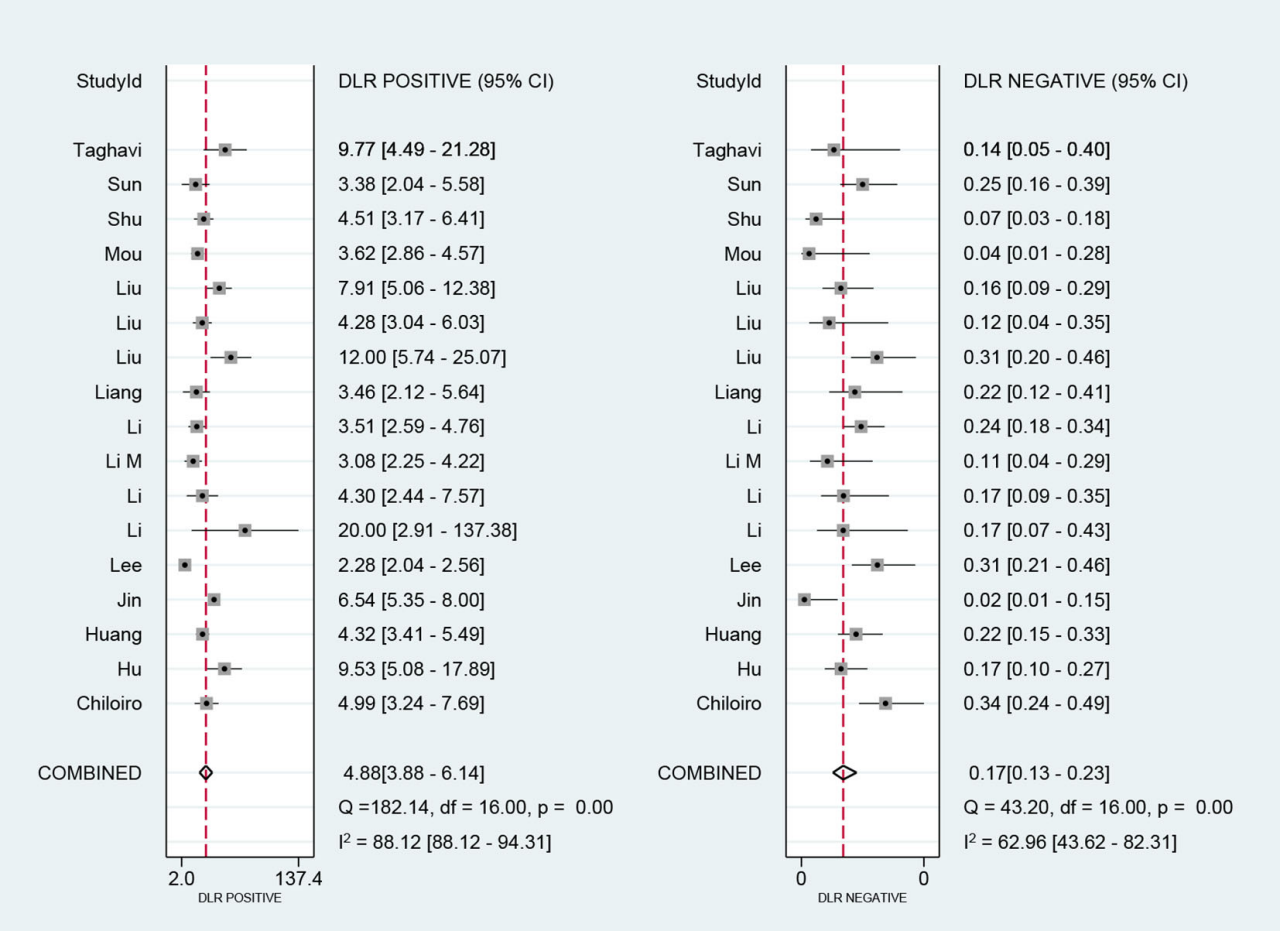
Combined sensitivity and specificity forest plot.

**Figure 4:**
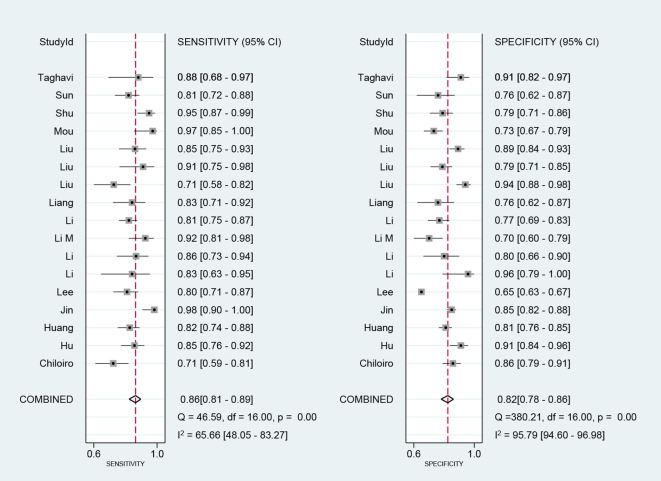
Forest plot for likelihood ratio after combination (LR+, LR-).

**Figure 5:**
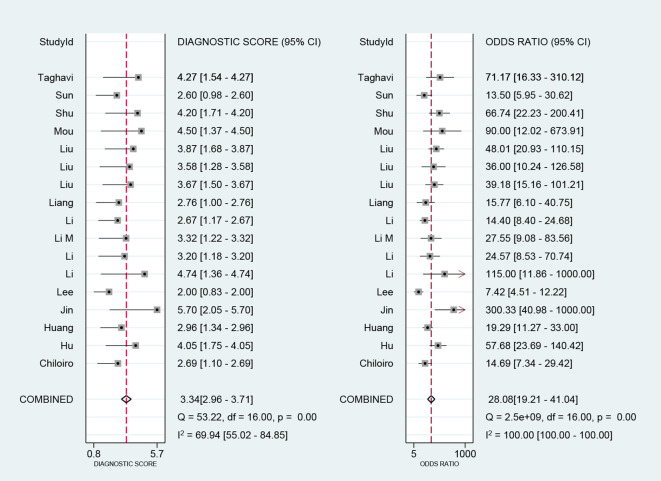
Forest plot for diagnostic odds ratio and diagnostic score after combination.

We performed a meta-analysis of the performance metrics of the predictive models. Diagnostic threshold analysis showed no significant threshold effect (Spearman correlation coefficient = 0.429, p = 0.13), but the results indicated a high degree of heterogeneity (Q = 34.4 with 2 degrees of freedom, p = 0.00; I^2^ = 94, 95% CI: 89- 99), for which a random-effects model was used to combine effect sizes. The pooled sensitivity and specificity were 0.86 (95% CI: 0.81-0.89) and 0.82 (95% CI: 0.78-0.86), respectively. The pooled diagnostic ratio, diagnostic score, positive likelihood ratio, and negative likelihood ratio were 28.08 (95% CI, 19.21-41.04), 3.34 (95% CI, 2.96-3.71), 4.88 (95% CI, 3.88-6.14), and 0.17 (95% CI,0.13-0.23), respectively (**Figures 3**–**5**).

**Figure 6:**
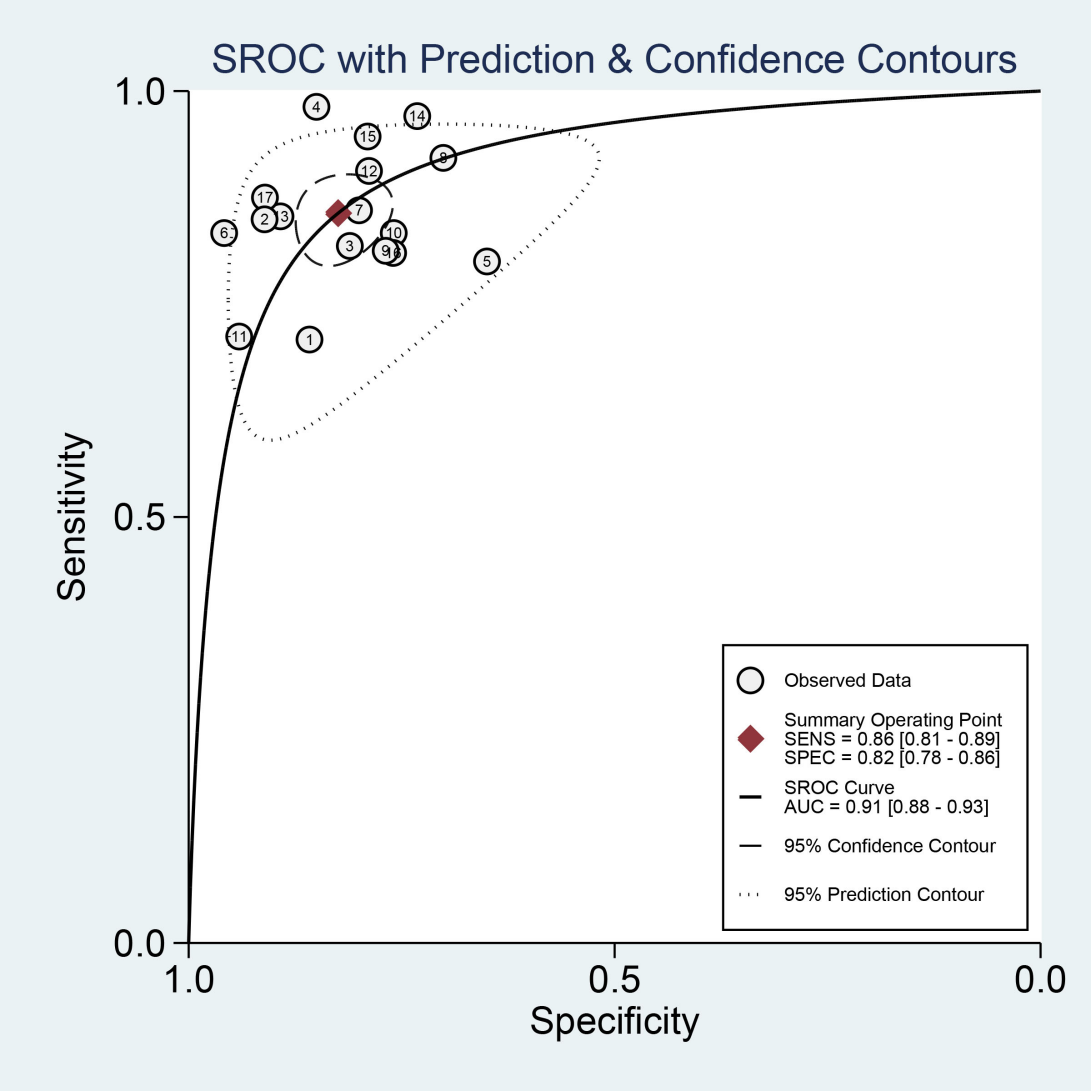
Summary receiver-operating characteristic and the area under the curve after combination.

In addition, we plotted sROC curves to evaluate the imaging model’s performance based on the AI algorithm in predicting distant metastasis of colorectal cancer (**Figure 6**). The results showed that the AI algorithm-based imaging model performed well in predicting distant metastasis of colorectal cancer with an overall AUC of 0.91.

To determine the source of heterogeneity, we performed a meta-regression analysis. **Table 4** shows the results of the meta-regression analysis, according to which our algorithm for considering the duration of follow-up, the site of metastasis (bone, peritoneal metastasis), and the lasso-constructed model were the sources of heterogeneity (p-value less than 0.05 for all). Our subgroup analysis showed that models based on large sample sizes had higher specificity (83% vs. 82%, p-value = 0.00). Regarding imaging modalities, ultrasound had higher sensitivity than other imaging modalities (97% vs. 85%, p-value = 0.04), and MR had higher specificity (85% vs. 80%, p-value = 0.00). Validation of the model using cross-validation had higher specificity (83% vs. 82%, p-value = 0.00), and validation of the model by other methods had higher sensitivity (85% vs. 84%, p-value = 0.00). Models that predicted (e.g., liver and lung metastases) but not multiple metastases, non-bone, or peritoneal metastases had higher sensitivity (p-value < 0.05), and models that predicted lung metastases had higher specificity (p-value < 0.05). In addition, studies using lasso-constructed models had higher sensitivity (p-value = 0.02) than those using other methods, whereas using other methods, non-SVM, LR, and Lasso-constructed models had higher specificity (p-value = 0.00).

**Table 4:** Subgroup analysis in combined model studies.

Variable		n	Sensitivity	P1	Specificity	P2	Joint model analysis		
							LRT chi^2^	P-value	I^2^
Country	china	14	0.85 (0.82- 0.89)	0.81	0.82 (0.78-0.86)	0.05	3.38	0.18	41
	Others	3	0.71 (0.54-0.88)		0.86 (0.74-0.98)				
Research approach	Retrospective	16	0.85 (0.81-0.89)	0.07	0.83 (0.79-0.87)	0.11	1.52	0.47	0
	prospective	1	0.81 (0.66-0.96)		0.76 (0.57-0.96)				
Sample size	>150	11	0.86 (0.81-0.90)	0.00	0.83 (0.78-0.87)	0.00	0.04	0.98	0
	≤150	6	0.86 (0.79- 0.92)		0.82 (0.74-0.89)				
Datasource	Multicenter	3	0.83 (0.73- 0.93)	0.03	0.85 (0.77- 0.93)	0.00	0.41	0.82	0
	Single center	14	0.85 (0.81- 0.89)		0.82 (0.78- 0.86)				
Imaging mode	CT	8	0.84 (0.79- 0.90)	0.00	0.81 (0.75-0.88)	0.00	0.32	0	0
	MR	8	0.83 (0.77-0.89)	0.00	0.85 (0.80- 0.89)	0.00	1.46	0.48	0
	Ultra	1	0.97 (0.91-1.00)	0.04	0.73 (0.56- 0.90)	0.01	4.72	0.09	58
Validation Methods	train-test split	4	0.85 (0.80- 0.91)	0.00	0.82 (0.77-0.88)	0.00	0.17	0.92	0
	external validation	3	0.82 (0.74- 0.90)	0.00	0.83 (0.75- 0.91)	0.00	1.22	0.54	0
	Cross-validation	10	0.84 (0.78- 0.90)	0.00	0.83 (0.77- 0.89)	0.00	0.71	0.70	0
Follow-up	>24months	5	0.82 (0.77- 0.88)	0.00	0.83 (0.76-0.89)	0.01	87.83	0.00	98
	≤24 months	4	0.83 (0.77- 0.89)		0.83 (0.74- 0.91)				
Distant metastatic site	multiple	5	0.81 (0.74-0.87)	0.00	0.85 (0.80- 0.90)	0.00	2.32	0.31	14
	hepatic	13	0.85 (0.80- 0.89)	0.00	0.81 (0.76- 0.85)	0.00	2.47	0.29	19
	pulmonary	3	0.85 (0.76- 0.93)	0.00	0.87 (0.80- 0.94)	0.01	2.22	0.33	10
	bone	2	0.71 (0.53- 0.90)	0.01	0.94 (0.89- 1.00)	0.98	6.58	0.04	70
	peritoneal	1	0.71 (0.53-0.90)	0.01	0.94 (0.89- 1.00)	0.98	6.58	0.04	70
AI algorithm	LR	10	0.83 (0.78-0.88)	0.00	0.82 (0.77- 0.88)	0.00	1.26	0.53	0
	Lasso regression	8	0.88 (0.84-0.91)	0.00	0.79 (0.74- 0.84)	0.00	7.37	0.03	73
	SVM	6	0.82 (0.75-0.89)	0.00	0.79 (0.72-0.87)	0.00	3.38	0.18	41
	others	12	0.81 (0.77-0.86)	0.00	0.84 (0.80- 0.89)	0.00	4.93	0.08	59

AI, artificial intelligence; CT, computed tomography; LR, logistic regression; Lasso, least absolute shrinkage and selection operator; MRI, magnetic resonance imaging; SVM, support vector machine.

### Fagan nomogram analysis

**Figure 7:**
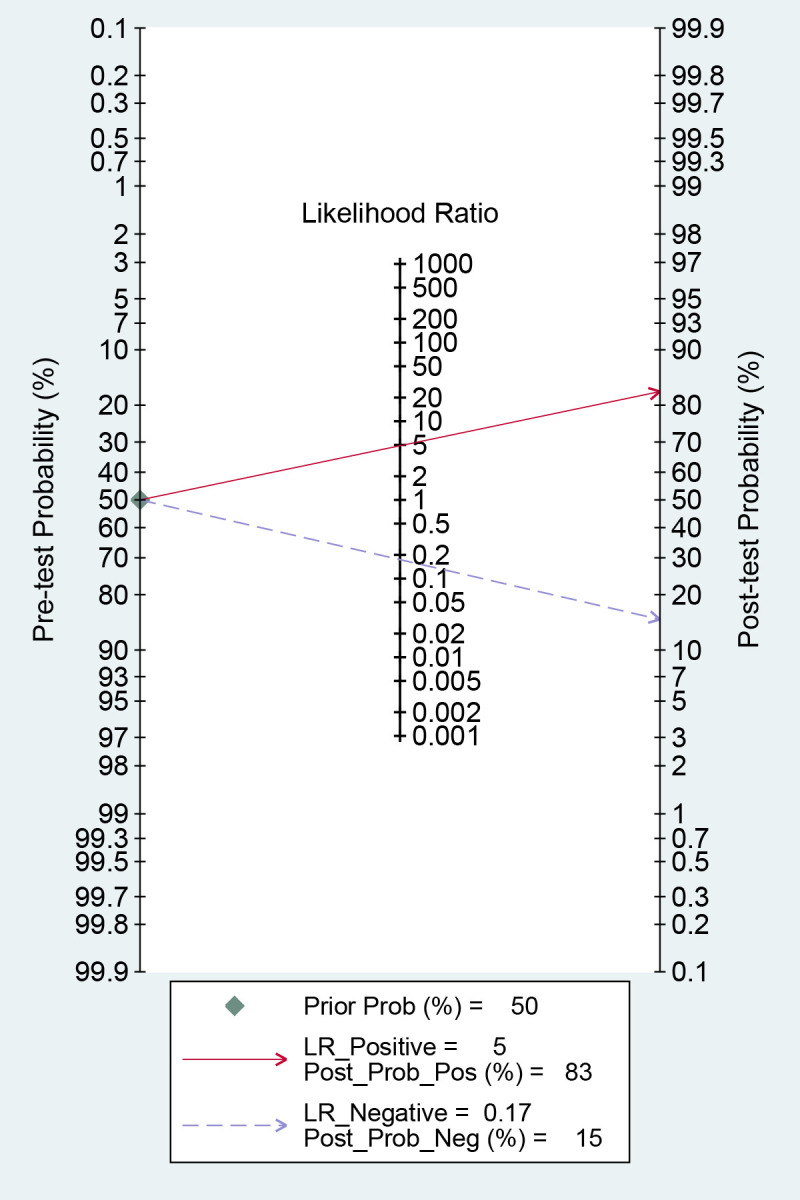
Fagan plots for assessing the clinical utility of models.

The AI-based imaging model could increase the post-test probability of predicting metastasis with a PLR of 5 from 50% to 83% when the pre-test was positive. When the pre-test was negative, the NLR was 0.17, and the post-test probability was 15% (**Figure 7**). These findings suggest that AI models are helpful in clinical practice.

### Publication bias and sensitivity analysis

**Figure 8:**
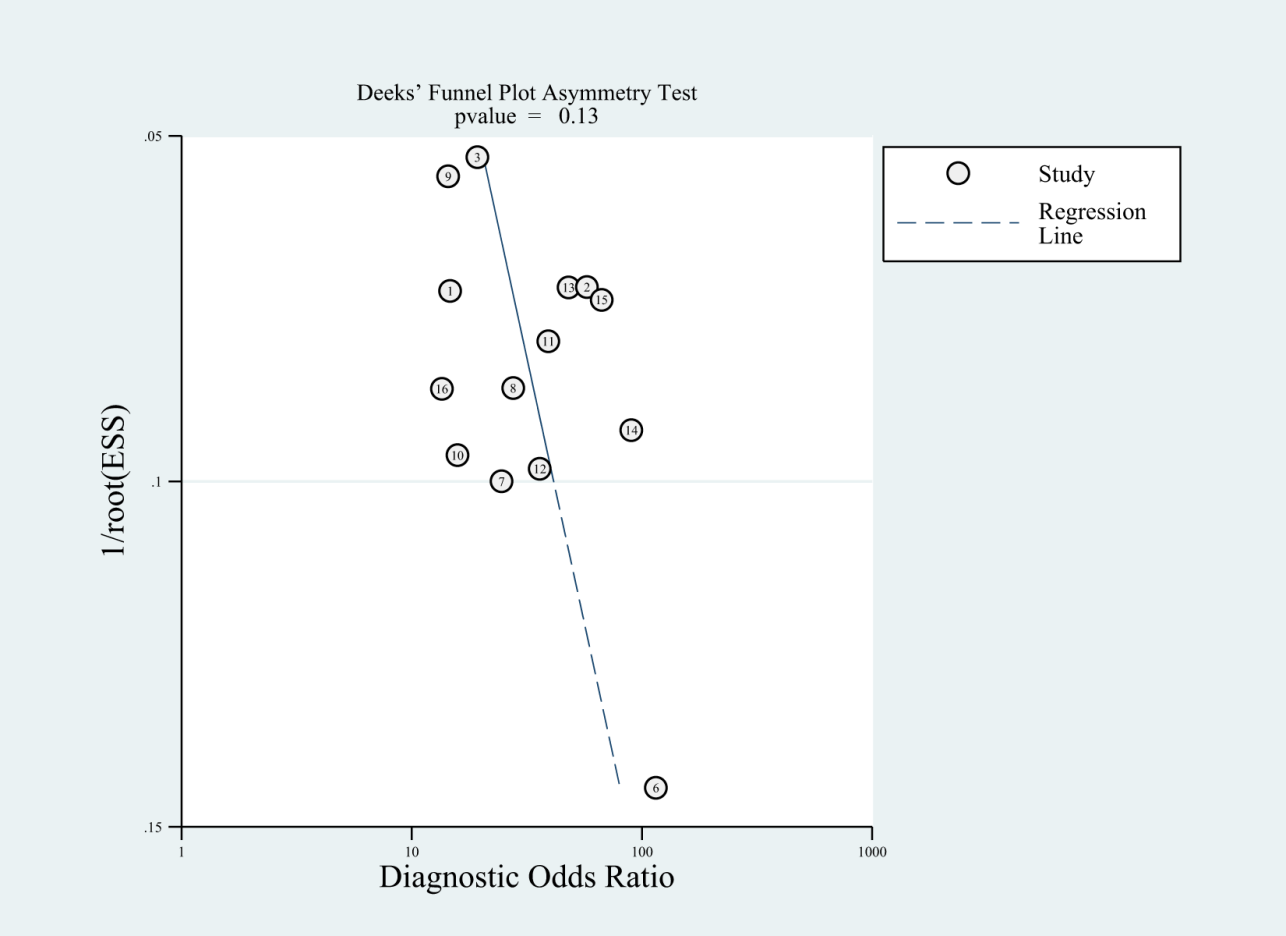
Deeks’ funnel plot with superimposed regression line. the funnel plot asymmetry test revealed no publication bias (P-values > 0.10).

**Figure 9:**
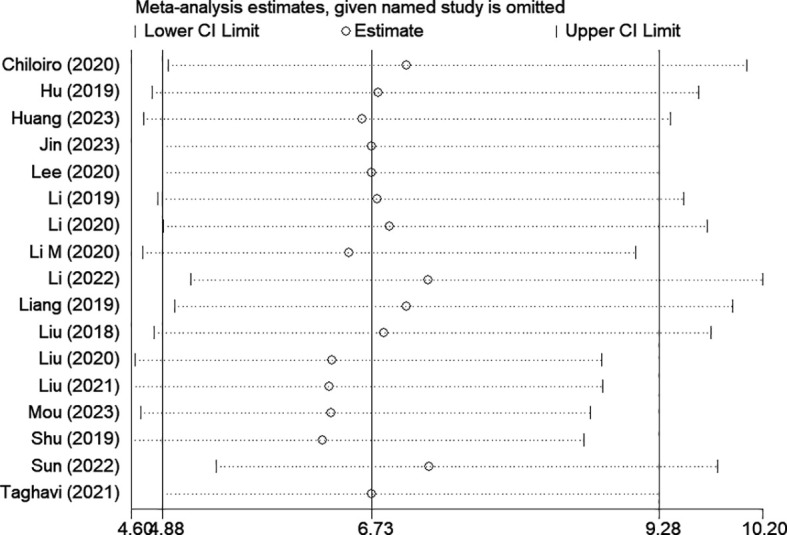
Sensitivity analysis.

Among the included studies, Deek’s test was used to investigate potential publication bias; however, the funnel plot asymmetry test showed no significant publication bias (p-value = 0.13) (**Figure 8**). When conducting the meta-analysis, we also performed a sensitivity analysis (**Figure 9**), which showed that the point estimates of the combined effect sizes after deleting a particular study fell between the 95% confidence intervals of the total combined effect sizes, indicating the stability of the findings.

## Discussion

This study investigated the value of artificial intelligence-based imaging data in predicting distant metastasis of colorectal cancer. The results showed satisfactory diagnostic accuracy with an overall AUC of 0.91 and pooled sensitivity and specificity levels of 86% and 82%, respectively.

In clinical practice, radiologists’ utilization of medical imaging and analysis of these images play a crucial role in detecting diseases. Due to the emergence of artificial intelligence, medical image analysis has become an up-and-coming field of study. A recent systematic evaluation demonstrated comparable performance between deep learning models and healthcare professionals in disease detection through picture analysis (34). The deep learning models exhibited a combined sensitivity of 87% and specificity of 92.5% in the analyzed investigations, whereas healthcare experts had a sensitivity of 86.4% and a specificity of 90.5%. This highlights the considerable potential of AI approaches in disease identification. Artificial intelligence employs sophisticated mathematical and computer algorithms to identify potential connections between characteristics and outcome variables (35, 36). These algorithms can forecast and enhance particular patient responses using existing data when applied to medicine. AI-based medical image analysis has demonstrated notable accuracy in predicting potential distant metastases with high sensitivity and specificity. While the current quality of AI studies is not yet adequate for routine clinical use, these findings indicate that AI-based medical images may be able to identify patients at high risk of developing distant systemic metastases after radical resection. Consequently, numerous researchers are endeavoring to utilize artificial intelligence (AI) in personalized medicine to enhance disease detection, therapy selection, and results (37). Staal et al. (38) examined 40 papers focused on colorectal cancer in their systematic review. They determined that artificial intelligence (AI) has demonstrated encouraging outcomes in predicting therapy response and long-term prognosis survival for this kind of cancer. Nevertheless, the authors recognized that a significant drawback of the mentioned studies was the heterogeneity of the included studies, specifically the various imaging techniques used to examine colon and rectal cancer. This indicates the necessity for careful consideration before implementing artificial intelligence results in clinical practice.

Likelihood ratios and post-test probabilities are valuable in determining the presence of distant metastases in patients with positive or negative test findings. Based on our study, a positive likelihood ratio of 5 means that the model is 5 times more likely to accurately identify a positive result than incorrectly identify a positive result. This leads to a post-test probability of a positive result of 83%. Similarly, a negative likelihood ratio value of 0.17 suggests that the model is 0.17 times more prone to incorrectly predicting a negative result than correctly predicting a negative result, resulting in a 15% chance of a pessimistic prediction. These findings additionally indicate that the use of AI-based imaging is precious in evaluating the presence of distant metastases in colorectal cancer.

In our study, we observed significant heterogeneity among the included studies. However, a threshold effect test measured by Spearman’s correlation coefficient indicated that a threshold effect did not cause the heterogeneity. Therefore, we performed meta-regression analyses for the source of data, sample size, follow-up time, imaging modality, model validation modality, transfer type, and different algorithms to explore possible sources of heterogeneity.

**Figure 10:**
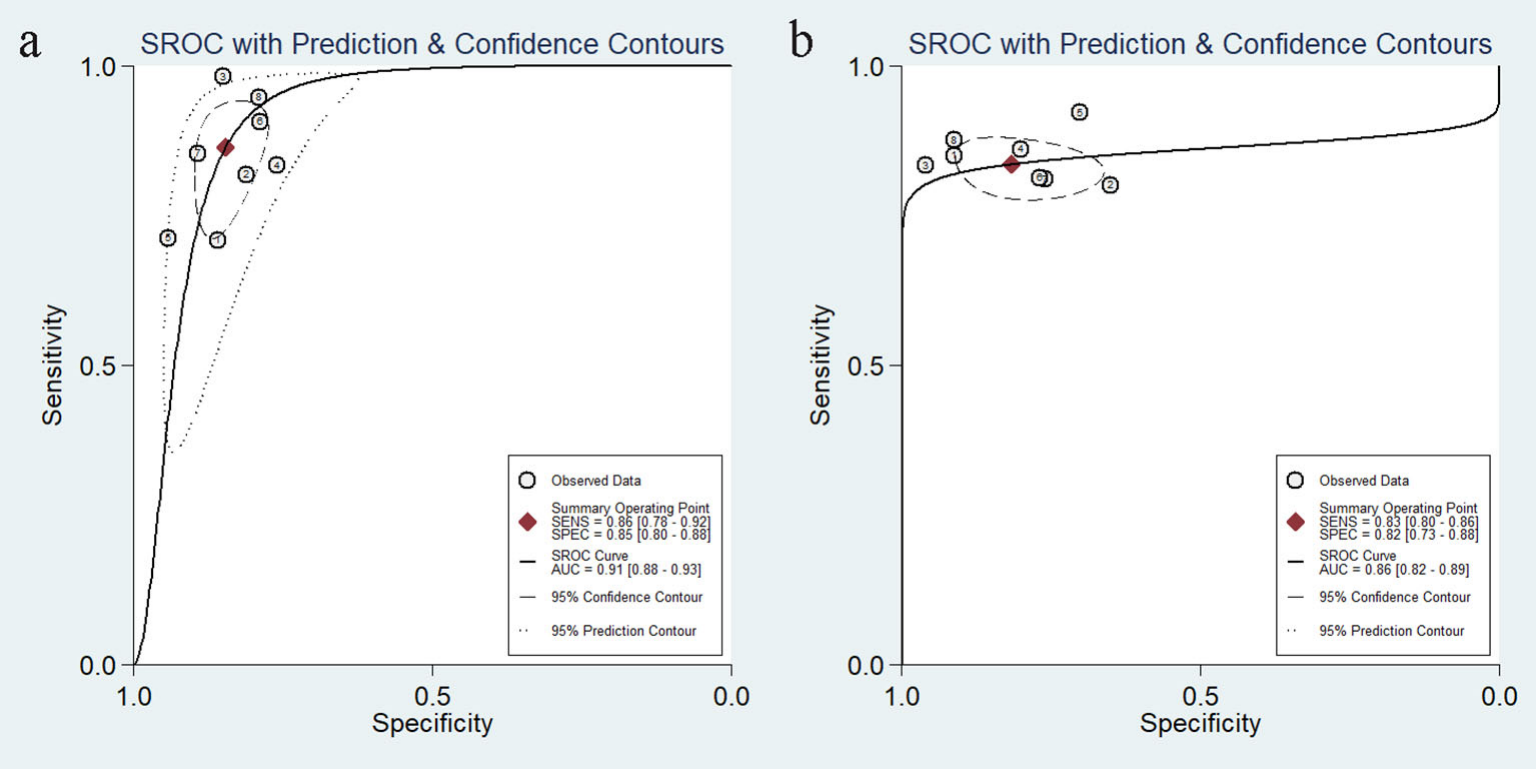
Summarized sROC curves for the model constructed based on MR images **(a)** and the model constructed based on CT images **(b)**. sROC, summary receiver operating characteristic.

We analyzed 17 studies in which CT and MR were the most commonly used imaging modalities, followed by ultrasound. This may be due to the disadvantages of ultrasound compared to CT/MRI, such as dependence on operator experience and patient condition, resulting in higher heterogeneity of ultrasound imaging modalities. In contrast, MRI can better characterize soft tissue features, atomic signal intensity, and lesion enhancement and provide more information about tissue function than CT. Our analysis showed that the ultrasound model based on AI algorithms has higher sensitivity than CT and MR, while MR has higher specificity with a pooled AUC of 0.91 (**Figure 10**). Our comprehensive literature search failed to identify any studies directly comparing the performance of different imaging modalities in predicting distant metastases, which may be because most of the literature reviewed consisted of different MRI sequences, with differences in sensitivity and specificity depending on the sequence selected. Therefore, prospective, large-scale, and multicenter studies may be needed to determine the superiority of one imaging modality over another.

In this analysis, the heterogeneity caused by different follow-up times was more pronounced, which may be because the longer the duration of follow-up, the higher the probability of distant metastasis. Whereas eight studies did not mention a precise follow-up time, we considered whether the lack of data caused higher heterogeneity. After deleting these eight studies and performing a subgroup analysis specific to follow-up time, we found significantly less heterogeneity between studies, while there was no statistically significant difference (I^2^ = 45, p=0.16).

**Figure 11:**
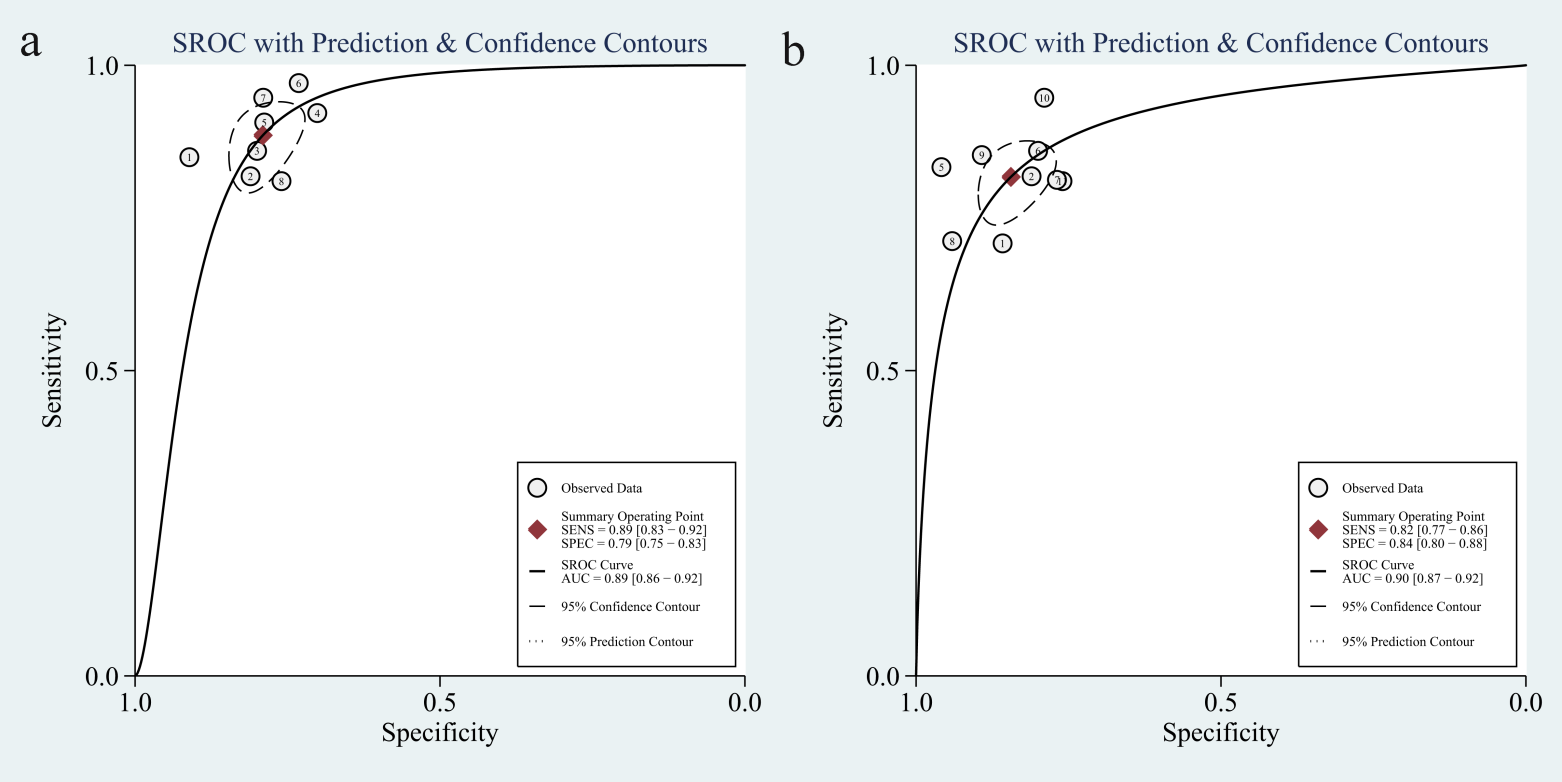
The pooled sROC curve of models constructed by lasso regression algorithm **(a)** and models constructed by others algorithm **(b)**. sROC, summary receiver operating characteristic.

The liver, peritoneum, lung, bone, and brain are the primary areas where colorectal cancer commonly spreads (39). The results of our study revealed a significant level of heterogeneity in predicting various types of metastases. Specifically, the two studies that focused on predicting bone and peritoneal metastases exhibited high levels of heterogeneity. This can be attributed to the limited number of studies on these specific types of metastases. The subgroup analysis revealed that the models predicting single metastasis, specifically liver and lung metastasis, showed higher sensitivity. Additionally, the models predicting lung metastasis exhibited the highest specificity. Model development can be achieved using many algorithms, including support vector machine, logistic regression, random forest, etc. Subgroup analyses were conducted on various AI algorithms, revealing that the model created using lasso had a higher sensitivity than the others. The pooled AUC for this model was 0.89 (**Figure 11a**). On the other hand, other algorithms, like convolutional neural networks, exhibited a relatively high specificity, with a pooled AUC of 0.90 (**Figure 11b**). In a meta-analysis of hepatocellular liver cancer, Zhang J et al. (40) conducted a study using AI-based imaging images to predict the features of MVI. Among the 13 studies, the model built with a convolutional neural network demonstrated high effectiveness in predicting MVI, with a pooled AUC value of 0.90. Nevertheless, it is essential to use caution when interpreting the findings of the subgroup analysis because the meta-analysis included a limited number of models.

In this study, we briefly analyzed and compared the artificial intelligence algorithms utilized in the literature and described the advantages and limitations of these models (**Supplementary Table S1**). The results indicate that the models constructed by most algorithms exhibit high sensitivity and specificity. Researchers frequently employ oversampling (SMOTE) when addressing imbalanced datasets, oversampling the minority classes within the training set, which involves augmenting the minority samples to approximate the number of positive and negative examples, followed by model training. Alternatively, the appropriate evaluation metrics are selected. For imbalanced datasets, the use of accuracy as an evaluation metric is potentially misleading; therefore, appropriate evaluation metrics, such as precision, recall, F1 score, and AUC, should be selected. For overfitting issues, cross-validation or regularization (L1/L2) (**Supplementary Table S2**) is often implemented.

Specific models that perform well on a particular task may not generalize to other tasks, and heterogeneity may be one of the main reasons specific models do not generalize to other tasks. The results showed high heterogeneity in our study, which is common in meta-analyses of imaging-based AI studies (41–44). However, these heterogeneities may still affect the generalizability of the results. According to the subgroup analysis, the sources of heterogeneity are various imaging modalities, different predicted metastatic sites, and different modeling approaches. Different medical scanners operate under different settings and datasets, and heterogeneity due to imaging modalities is mitigated by developing methods that can be validated on different types of images. Most colorectal cancers metastasize to the liver and lungs. Our results showed that only two articles were from patients with bone metastases, and one was from patients with peritoneal metastases. Moreover, the appearance of different metastatic tumors on imaging may differ. Therefore, this comparison is not ideal. This is still an open research area that requires further study, and different models may need to be designed for different metastatic tumors to obtain satisfactory performance. Despite the advances in AI-based medical imaging algorithms, there are still deficiencies in the different algorithms. In the case of different algorithms, these shortcomings include patient selection, image acquisition, a limited number of studies, and lack of uniform study protocols, which result in a wide range of sensitivity and specificity values, making it challenging to compare results. Future research should focus on validating AI-based algorithms in prospective studies, investigating the inner workings of the algorithms, developing interpretable AI models, integrating AI radiomics features with clinical data, and developing standardized methods for data collection and feature extraction.

In recent years, AI has demonstrated remarkable developmental momentum. If appropriately utilized, it may yield optimal outcomes across numerous application domains. AI has achieved unprecedented performance levels in learning to solve increasingly complex computational tasks, thereby becoming pivotal to the advancement of human society. The complexity of AI-driven systems is escalating, such that their design and deployment necessitate minimal human intervention. However, the decision-making processes of AI systems are often perceived as a ‘black box,’ with their internal operational mechanisms and decision rationales frequently remaining opaque. Consequently, eXplainable Artificial Intelligence (XAI), such as SHapley Additive exPlanations (SHAP) and Local Interpretable Model-agnostic Explanations (LIME), is widely considered a critical feature for the practical deployment of AI models. Its core objective is to elucidate the ‘black box,’ revealing how AI generates specific predictions or decisions, along with the underlying logic and rationale. Of the AI models assessed in this study, 13 employed intrinsically interpretable models, including linear regression and decision trees, while few studies utilized SHAP and LIME, a disparity that contrasts with the requirements for retrospective decision-making in clinical practice.

This study has several limitations. First, because this study was a systematic review of pooled data from multiple studies, it was inherently limited by the included studies. Most of the included studies were retrospective, inevitably leading to patient selection bias, and only three of the included studies used independent external validation cohorts to assess model performance, which limits comparisons in terms of predictive features and model robustness. Our ultimate goal is to apply the developed imaging model based on artificial intelligence algorithms to improve prognosis. On this basis, our model and estimation results should be generalizable to practice. However, most included studies used internal model validation, which is more prone to overestimation and lack generalizability. Therefore, prospective studies and more external validation are necessary to assess model performance on unseen data before applying the models to the clinic. Second, the heterogeneity among the included studies regarding imaging modalities and modeling methods should be addressed. The majority of studies were conducted within a single-center setting in China, and the patient recruitment from a single center constrained the generalizability and reproducibility of the findings. Furthermore, regional bias should be considered due to variations in disease backgrounds across different regions, countries, and races, which may diminish the generalizability of artificial intelligence models beyond China. It is recommended that future research incorporate multi-center studies across a broader range of countries. Finally, the majority of the included literature in this study provided limited quantitative assessment of model explainability and lacked comprehensive reporting on integration with existing clinical decision-making processes. Future research should incorporate the validation of XAI within the framework of model performance evaluation.

## Conclusion

In conclusion, Our study demonstrates that AI algorithms may accurately predict tumor metastasis in medical radiography. These algorithms exhibit high sensitivity and specificity, making them suitable for clinical use. The extensive use of this technology in clinical settings can help address the scarcity of medical resources, enhance the rate and precision of tumor metastasis identification, and consequently enhance patients’ prognosis. Nevertheless, it is imperative to recognize the necessity for additional rigorous study into the implementation of artificial intelligence in the field of medicine in order to advance clinical practice and establish standardized research protocols. Future research should prioritize prospective studies with more significant sample numbers and explore various imaging modalities. Additionally, it is essential to emphasize the quality of reporting, validate the external model, ensure generalization to actual clinical circumstances, and improve the reproducibility of results.

## Data Availability

The original contributions presented in the study are included in the article/**Supplementary Material**. Further inquiries can be directed to the corresponding author.
