# Supplementary material for: Diagnostic accuracy of artificial intelligence based on imaging data for predicting distant metastasis of colorectal cancer: a systematic review and meta-analysis
Source: Front Oncol. 2025 May 12;15:1558915. doi: 10.3389/fonc.2025.1558915 (PMC12104061; doi:10.3389/fonc.2025.1558915)
Supplement: Supplementary file 3 [file Table1.docx]

TableS1: Comparison of Artificial Intelligence Algorithms for Building Models

| **Algorithm** | **Strengths** | **Limitations** | **Accuracy** | **Sensitivity** | **Specificity** | **AUC** |
| --- | --- | --- | --- | --- | --- | --- |
| SVM | High-dimensional data classification is robust, and small samples perform well. | Sensitive to missing data, kernel function selection affects performance | 0.91 | 0.86 | 0.95 | 0.96 |
| LASSO | The model is highly interpretable and anti-overfitting. | Sensitive to relevant features, only linear relationship. | NR | 0.86 | 0.81 | 0.86 |
| PCA | Computationally efficient and suitable for exploratory analysis | Ignoring the discriminant information, the principal component interpretation is poor. | NR | 0.95 | 0.79 | 0.89 |
| RF | Anti-overfitting, feature importance analysis intuitive | Low interpretability and high computational complexity | 0．87 | 0.72 | 0.94 | 0.83 |
| LR | Strong interpretability and high computational efficiency | Linear hypothesis limits nonlinear relation modeling | 0.76 | 0.80 | 0.83 | 0.88 |
| CNN | Local feature extraction ability is strong, and image processing performance is excellent. | Large-scale annotation data is required, and the demand for computing resources is high. | 0.92 | 0.89 | 0.94 | 0.96 |
| GBDT | High prediction accuracy, strong robustness, support for nonlinear relationship and feature interaction, without feature engineering. | The training speed is slow, the parameter tuning is complex, the memory consumption is high, and the high-dimensional sparse data is weak. | NR | 0.78 | 0.82 | 0.85 |
| MLP | Strong expression ability, can approximate any complex function, automatic feature learning, high flexibility. | Small samples are easy to overfit, rely on data enhancement or regularization, and the difficulty of parameter adjustment is high. | NR | 0.70 | 0.67 | 0.78 |
| SCLF | It integrates the advantages of diversity, strong generalization performance, and supports the free combination of any base model and meta model. | The computational cost is high, and model stacking may lead to overfitting and extremely low interpretability. | NR | 0.78 | 0.82 | 0.84 |
| DT | Strong interpretability, non-parametric | Overfitting risk, ignoring feature interaction | NR | NR | NR | 0.95 |
| SMOTE | Relieve category imbalance and avoid oversampling repetition | High-dimensional data synthesis effect is poor, overlapping between classes. | NR | NR | NR | NR |
| KNN | Simple and easy to implement, no distribution assumption | Low computational efficiency, high-dimensional failure | 0.95 | 0.84 | 0.99 | NR |
| LD | The combination of dimension reduction and classification, small sample stability | Linear hypothesis limitation, normal distribution dependence | NR | 0.78 | 0.76 | NR |
| NB | Fast calculation speed, high-dimensional friendly | Unrealistic independence assumptions and poor probability calibration | NR | 0.70 | 0.72 | NR |
| ANN | Nonlinear modeling, automatic feature learning | Black box model, high demand for data and computing power | NR | NR | NR | 0.91 |
| ResNet | Residual connection, feature reusability, multi-task adaptation, and strong robustness | High hardware dependence, large number of model parameters, redundant structure and parameters, and overfitting of small data scenarios. | 0.93 | 0.86 | 0.89 | NR |
| ComBat | Cross-batch data correction, retain biological signals | Normal distribution assumption, batch label dependence | NR | NR | NR | NR |

AUC: area under the curve; CNN: convolutional neural network; DT : decision tree ; GBDT : gradient boosting decision tree ; KNN: k-nearest neighbor; Lasso: least absolute shrinkage and selection operator; LR：logistic regression；LD:linear discriminant ; MLP: multilayer perceptron ; NB:naive Bayesian ; NR:not reported;PCA:principal component analysis; RF:random Forest;SVM: support vector machine; SCLF:stacking classifier ; SMOTE: synthetic minority oversampling technique.

Table S2: Data processing information

| Studies | Data processing information |
| --- | --- |
| Li，2019 | Features are computed from gradient images using statistical (FoS) and textural (CoM and RLM) methods；After image features are extracted from preoperative routine CT data, the features are normalized using a z-score normalization method to achieve stable convergence, and the hyperparameters (kernel parameters and penalties) of the generated classifier are optimized； |
| Liang，2019 | The consistency checks, feature selection, model development, and statistical computations were performed and the graphics were prepared using the Python (https://www.python.org/) in Anaconda3 platform (https://www.anaconda.com) with Scikit-learn (https://scikit-learn.org/) and Matplotlib packages (https://matplotlib.org/); all radiomic features were standardized by removing the mean value and then dividing it by its standard deviation and transformed into feature values with a mean of zero and a standard deviation of one; The least absolute shrinkage and selection operator regression (LASSO) method was implemented to select the optimal features from the primary T2WI and VP features and the combined radiomic features incorporating the primary T2WI and VP sequences |
| Shu，2019 | Firstly, extracted texture features were standardized, which could remove the unit limits of the data of each feature so that the indexes of different units or orders could be compared and weighted. Then the feature dimensionality reduction was performed as follows. The analysis of variance (ANOVA) and Mann-Whitney U test (MW) dimensionality reduction were performed, and then the correlation test was calculated to reduce data redundancy. The software calculated the paired correlation between each two of the features. If the Spearman correlation coefficient was greater than 0.9, which showed that the two features were highly correlated, one of them was removed. |
| Lee，2020 | feature extraction on the images was done by utilizing a pre-trained convolutional neural network, VGG16; preprocessed the extracted imaging features based on the significance of association with 5YLM rate by performing univariate logistic regression analysis; the subsets of imaging features that passed the suggestive significance level (P < 0.01) were used for further analyses; principal component analysis (PCA) was performed for reducing the feature dimensionality, and  this generated new sets of features; At the input stage to the machine learning methods, we further standardized either the clinical features or the PC-transformed image features, by znormalization for each feature to have a mean of zero and unit variance; (splitting the data set into a training set and a test set; SMOTE followed by down-sampling; a five-fold cross validation to find the optimal parameters; and performance evaluation on the test set) for 100 iterations; |
| Li，2020 | (I) DICOM file preprocessing and (II) data processing. Its main functions include the following: (I) removing personal information from DICOM files and optimizing ROI extraction; (II) extracting either fewer or more radiomics features based on the radiologists’ needs; (III) acquiring the radiomics features signature using different algorithms; (IV) building and saving high-accuracy machine learning models by automatically selecting the optimal parameters; independently select the radiomics feature signature from the cross-validation set only to ensure that the test set is unaffected；use gradient feature selection to evaluate the features of each CT image；Features are selected when the t-test results are P<0.05 based on the hypothesis. Then, these features are further filtered by LASSO with 10-fold cross-validation. We select the α value in which the mean square error is the smallest. Using the α value, we obtain the radiomics feature signature and feature coefficients. |
| Taghavi，2021 | Prior to feature extraction, images were normalized to reduce influence of differences in CT scan protocol between three centers and all images interpolated to isotropic voxels of 1 mm3 using a B-Spline interpolator. In addition, for textural features, gray values were quantized using a fixed bin width of 2; Feature extraction was done using 3D extraction implemented in PyRadiomics. inter-correlated remaining features were removed: if the absolute Pearson correlation for each pair of features F1 and F2 was 0.9 or higher across all patients, F2 was removed; features were standardized with zero mean and unit variance; a wrapper method was used to find the best set of features |
| Li，2022 | An L2-based normalization method was used to rescale each type of radiomics feature. Then, the recursive feature elimination (RFE) method was applied to reduce the dimensionality of feature spacing and remove the redundant image features; synthetic minority oversampling technique (SMOTE) was used to increase the number of minority samples in the dataset. |
| Sun，2022 | Te data are dimensionally reduced by methods such as the rank sum test (ANOWA + MW), correlation analysis, and Lasso, using LR,LD, KNN, NB, DT, and SVM classifiers classify the extracted texture features. |
| Jin，2023 | linear method is used for resampling; Gaussian 0.50 is used for noise removal; MR bias field correction is adopted to eliminate the stray intensity change caused by the non-uniformity of magnetic field and coil; Intensity standardization adopts gray discretization, and the expected minimum value and maximum value are 0.015 and 0.255,respectively；AK（Artifical Intelligence Kit,V3.2.0R version） software to capture texture features; balance the positive/negative samples by reducing the sampling, and use Min Max to normalize the feature matrix. At the same time, we use Pierce correlation coefficient to reduce the dimension of the data,and the eigenvectors of the transformed eigenmatrix have independent features; use recursive feature elimination algorithm to select features and sort them. |
| Li M，2020 | Before selecting features, the redundant features needed to be eliminated. When the Pearson correlation coefficient of any two features was higher than 0.6, one of them was selected at random, and the rest could be treated in the same manner. Then, the least absolute shrinkage and selection operator (LASSO) regression analysis was performed to identify the most useful prognostic features; z-score transformation was used to standardize all selected features |
| Liu，2021 | used the primary cohort to fine-tune ResNet18 pre-trained on ImageNet. |
| Liu，2018 | the best-performing features based on the univariate statistical tests including analysis of variance (ANOVA), Kruskal-Wallis test, univariate logistic regression analysis, and Pearson correlation analysis with a Pearson correlation coefficient of 0.9 were selected; a random forest algorithm was applied to select the most informative features that can contribute to the overall classification between the two groups in the primary dataset. |
| Chiloiro，2020 | Features selection was performed using a 5-folds crossvalidation method；The selected features were analyzed for correlation based on Pearson's correlation coefficient, and only features with less than 30% correlation were selected；The up-sampling method was used to handle the outcome class imbalance. |
| Hu，2019 | the least absolute shrinkage and selection operator (LASSO) algorithm was used to select the predictive radiomics feature in the training cohort；The tenfold crossvalidation was implemented to avoid over-fitting； |
| Liu，2020 | applied a variance threshold method to reduce the features, with the threshold setted to 0.8. On this basis, the least absolute shrinkage and selection operator (LASSO) method was used to screen the optimal features to predict the SLM. |
| Huang，2023 | The imaging features were normalized by using min–max normalization；two-sample t test was initially used to detect the differences between patients with diverse outcome according to their 3- year DFS for each feature,and features with p < 0.05 were used for further analysis；the least absolute shrinkage and selection operator was then utilized to further select robust and nonredundant features, and penalty parameter tuning was performed by 10-fold cross-validation；performed additional analysis by using ComBat to correct statistical variation due to batch effects |
| Mou，2023 | utilized z-score methods to perform radiomics features normalization in training and validation cohorts; The Wilcoxon test was performed to pick out the radiomics features associated with SLM in RC in the training cohort; performed least absolute shrinkage and selection operator (LASSO) with five-fold cross-validation to find the optimal feature subset, which is inclined to eliminate highly related features, and then evaluated the performance of multivariate regression models by multiple λ values with fewer features. |
